# Plant ER geometry and dynamics: biophysical and cytoskeletal control during growth and biotic response

**DOI:** 10.1007/s00709-016-0945-3

**Published:** 2016-02-10

**Authors:** Lawrence R. Griffing, Congping Lin, Chiara Perico, Rhiannon R. White, Imogen Sparkes

**Affiliations:** 1 Biology Department, Texas A&M University, 3258 TAMU, College Station, TX 77843 USA; 2 Mathematics Research Institute, Harrison Building, University of Exeter, Exeter, EX4 4QF UK; 3 Biosciences, CLES, Exeter University, Geoffrey Pope Building, Stocker Rd, Exeter, EX4 4QD UK

**Keywords:** Endoplasmic reticulum, Movement, Actin, Myosin, Microtubules

## Abstract

The endoplasmic reticulum (ER) is an intricate and dynamic network of membrane tubules and cisternae. In plant cells, the ER ‘web’ pervades the cortex and endoplasm and is continuous with adjacent cells as it passes through plasmodesmata. It is therefore the largest membranous organelle in plant cells. It performs essential functions including protein and lipid synthesis, and its morphology and movement are linked to cellular function. An emerging trend is that organelles can no longer be seen as discrete membrane-bound compartments, since they can physically interact and ‘communicate’ with one another. The ER may form a connecting central role in this process. This review tackles our current understanding and quantification of ER dynamics and how these change under a variety of biotic and developmental cues.

**Electronic supplementary material:**

The online version of this article (doi:10.1007/s00709-016-0945-3) contains supplementary material, which is available to authorized users.

## Introduction

**Fig. 1:**
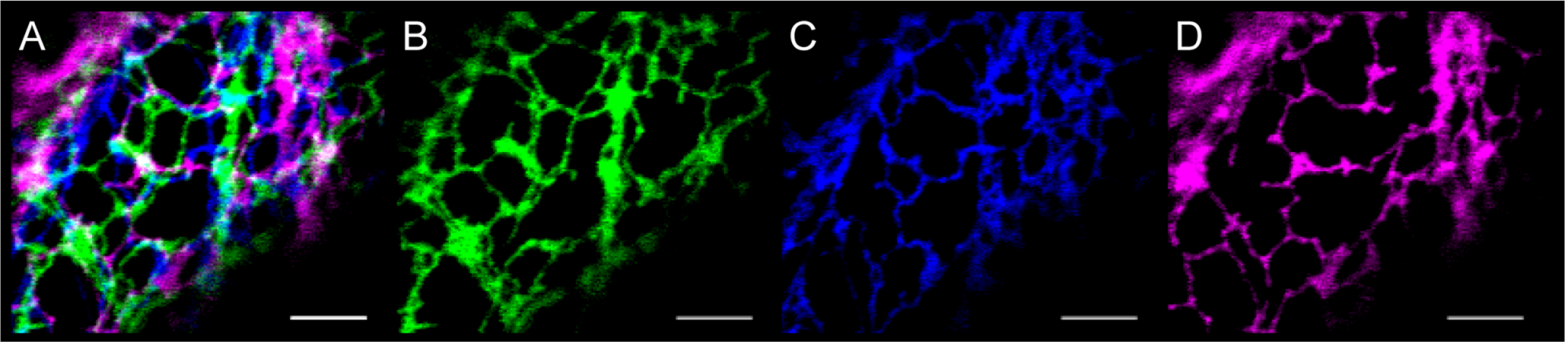
The cortical ER in plant cells is highly dynamic. Confocal image of tobacco leaf epidermal cells expressing an ER luminal marker (GFP-HDEL). **a** Overlay of three consecutive images taken 15 s apart where *white* indicates GFP-HDEL fluorescence at all three time points: **b** *green* = 0 s, **c** blue = 15 s and **d** magenta = 30 s. Images taken from Supp. Movie 1. *Scale bar* = 5 μm

The endoplasmic reticulum (ER) is the starting point for the secretory pathway and can be viewed as a biosynthetic hub within the cell. It consists of a large interconnected network of flattened cisternal regions (also referred to as sheets) and tubules, which form three-way junctions and blunt ends, all of which undergo drastic remodelling within a short time frame (see Fig. 1 and Supp Movie 1). Morphology is linked to the cells’ secretory capacity and developmental stage (Ridge et al. 1999; Stephenson and Hawes 1986), and components that affect ER geometry are affected by external stresses (Lee et al. 2012). Early observations of ER movement in unstained (Lichtscheidl and Url 1987) and stained cells (Lichtscheidl and Url 1990; Quader and Schnepf 1986) and those expressing an ER-targeted green fluorescent protein (GFP) fusion (Ridge et al. 1999) all highlighted the dynamic nature of the network in plant cells. This review will first introduce the current methods in assessing and quantifying ER movement; then, it highlights the molecular factors that control movement and, finally, how this movement changes during plant development and interaction with the environment. The components that drive movement and morphological form include the reticulons, associated proteins such as root hair defective 3 (RHD3) and the actin cytoskeleton. Since reticulons and RHD3 have been reviewed elsewhere, they will not be covered here (Stefano et al. 2014a; Sparkes et al. 2011).

## Quantifying ER geometry and dynamics

The ER network can drastically remodel within a short time period. Remodelling can be split into two areas: (1) remodelling of the surface of the ER membrane itself (referred to as surface flow) and (2) global remodelling of the structure resulting in alterations in total morphology.

### Surface flow

Surface flow is not necessarily directly linked to changes in morphological form. This has been shown through photobleaching and photoactivation experiments that quantify how a fluorescently labelled ER membrane probe (e.g. calnexin) migrates within the ER membrane (Runions et al. 2006; Sparkes et al. 2009a). The membrane probe shows lateral mobility in the actively remodelling membrane. Upon stopping movement of the entire network (through treatment with latruculin b, see later), probe mobility is significantly reduced but is still able to diffuse within the membrane (Runions et al. 2006; Sparkes et al. 2009a). It is unclear what other components, such as molecular crowding or corralling from interaction with cytosolic components, may be controlling this motion. Future efforts to look at lateral mobility of functional proteins, rather than probes which contain a transmembrane domain, will allow a clearer understanding of the interplay between surface flow and remodelling of the entire network itself and the functional role of this motion. It is interesting to note that whilst the ER is tethered to the plasma membrane at membrane contact sites (MCSs) and that the molecular components involved in this process have both actin and microtubule dependency, then this raises the interesting possibility that ER mobility may be influenced through signalling from the plasma membrane (PM), and the effects observed upon actin depolymerization may be mediated through microtubule-dependent processes (see later). The view that modelling is driven solely by actin-dependent processes is therefore relatively simplistic.

### Global morphological change

Within the ER network, there are relatively static and highly dynamic elements (Griffing 2010; Sparkes et al. 2011; Sparkes et al. 2009a). Static elements refer to regions which do not move within a given time period, and these can be fairly stable such as MCSs with the plasma membrane or can change into dynamic regions. Dynamic regions refer to tubule growth (i.e. extend), shrinkage (i.e. retract), lateral sliding at its connection points to form three-way junctions and closed polygons, changes between tubular and cisternal forms and changes in size and morphology of such (see Fig. 1). All of these are further complicated by fast movement within the cytoplasmic streams. In this complex environment, how do you begin to start to quantify the multifaceted network?

As with any complex problem, the starting point is to isolate the components and start with the one which is easiest to address. The most convenient starting point is therefore to identify and quantify changes in the static regions within the network. This has been carried out through persistency mapping and has pulled out not just static nodes (0.1–03 μm^2^) but also static tubules and cisternal regions (more than 0.3 μm^2^) within the network (Sparkes et al. 2009a). The same static nodes were also identified through optical trapping experiments whereby physical attachment between the ER and Golgi allowed the users to pull and remodel the ER through micromanipulation of the Golgi stack (Sparkes et al. 2009b). By pulling and wrapping the ER around these static islands or nodes, it was possible to observe stable association and subsequent generation of three-way junctions as the ER was extended away from the static nodal points. Three-way junction formation is therefore likely a biophysical principle of extension of a tubular membrane from fixed points.

To begin to quantify the biophysical components/nature that drive dynamic network formation, a skeletonized version of the network from the live cell imaging data was generated, and then, the components within the resulting skeleton were quantified in terms of movement of ER junctions, branching angles of junctions and the connection between persistent points and other nodes (Lin et al. 2014). Nodes within the resulting skeleton refer to either persistent points, ends of ER tubules and ER junctions. Here, the data on individual networks was derived from both relatively ‘static’ networks (latrunculin b treated) and an unperturbed native dynamic network (Lin et al. 2014). These basic measures of the system were used to model formation of, and understand the biophysical constraints on, the interconnected network and has contributed somewhat towards modelling the dynamic network itself. Commonalities between ER network formation and network patterns present in nature led the authors to assess whether the network may form and tend towards limiting its entire length, and by doing so, it may therefore optimize its entire length (Lin et al. 2014). This type of optimization problem can be studied using a minimal spanning tree theory. Here, the ER would generate the shortest length possible by connecting or wrapping itself around the static nodes. Analysis revealed that the resulting ER network locally tends towards a Steiner network, which is similar to a minimum spanning tree, but allows for additional nodes to be added. These additional nodes are the three-way junction points that form as a result of extending the ER tubules from static nodes. The Steiner network analysis also allows for polygon formation. Moreover by adding constraints on the allowable size of angles between tubules forming and extending from a node, the unique minimal network between observed ER nodes shows a similar network structure to that of the observed native ER networks in vivo (Lin et al. 2015). Furthermore, by taking all of these parameters into consideration, and then modelling network formation around static nodes and three-way junction points that undergo Brownian motion, has again allowed for a certain degree of accuracy in simulating ER network formation. In conclusion, simulations of ER formation based on measured parameters and graph theory indicate that the network does undergo a certain degree of length optimization and that both random (Brownian motion) and deterministic (such as motor based) forces affect these processes (Lin et al. 2014, 2015; Lemarchand et al. 2014). In addition, these studies have also provided a first-order approximation on ER tubule tension by considering the force balance between tension, Stokes drag force and Brownian force (Lin et al. 2014). As the network is complex, these types of studies on ER network dynamics have so far been limited to tubular regions of the ER network and monitoring the dynamics of the relatively static network (i.e. Brownian motion). However, the native dynamic network (i.e. not treated with latrunculin b) also shows a Steiner network-like configuration (Lin et al. 2014).

Persistency mapping and modelling dynamical elements of the network have therefore considerably furthered our understanding and quantification of the ER network. Future studies will need to take into consideration both tubule and cisternal elements of the ER and active remodelling and cytoplasmic streaming to provide a more comprehensive dynamic model of the entire system. Note, other types of models have been proposed to describe how (1) molecular components that ‘shape’ the ER, such as reticulons, induce physical curvature and (2) the energy requirements for network formation in other systems (Schweitzer et al. 2015; Shemesh et al. 2014; Terasaki et al. 2013). It is likely that energy considerations for ER network formation are not species specific as they are governed by biophysical principles. However, the relative levels of tubules to cisternae, and therefore the regulation of the molecular components that control these morphological changes, are likely to be under species (and tissue) specific regulation.

## Molecular control of ER movement

The ER is a highly mobile network with tubules growing (1–1.5 μm/s) at rates similar to that of actin polymerization. The previous section highlighted the inherent issues of quantifying such a dynamic network and the progress made to date. Identifying molecular components that drive and control ER dynamics are based on being able to quantify the effect they have on ER dynamics. At present, this is somewhat limited to identifying changes in the static regions of the ER network or large-scale changes which reduce the overall movement of the entire network. Therefore, it is difficult to determine and quantify the role of molecular components that drive *specific* elements of ER dynamics, for example tubule growth, shrinkage and polygon formation.

Prior to developing quantification platforms for the ER network, large-scale changes in network dynamics were inferred from pharmacological studies which perturb the cytoskeletal network itself. By effectively removing the cytoskeletal scaffold which the ER network utilizes for movement, it was possible to determine that actin, rather than microtubules, play a major role in ER dynamics in higher plants (Knebel et al. 1990; Liebe and Menzel 1995). However, studies from Characean algae (Foissner et al. 2009) infer that cortical microtubules may also have a role during certain developmental stages and control the density of polygons per unit area: the mesh size. Cortical microtubules may also control slow rates of tubule extension and provide branch points in the cortical ER in Arabidopsis (Hamada et al. 2014).

**Fig. 2:**
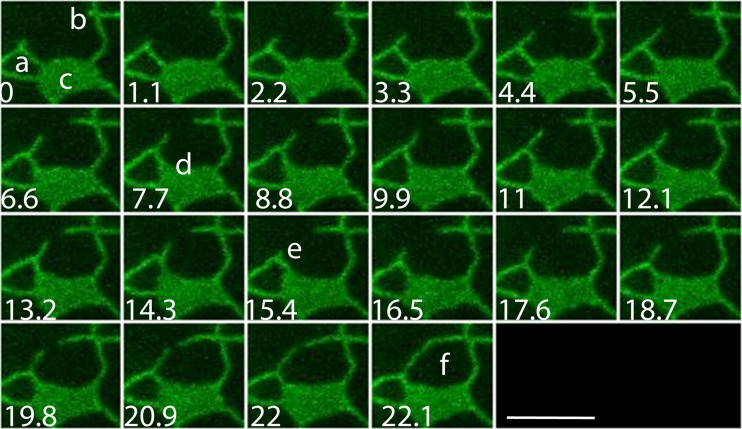
Montage of tubule fusion in myosin *xi-k*, *xi-1*, *xi-2* Arabidopsis triple mutant. *Lower left numbers* are in seconds. **a** Polygon from which a new branch is forming at the top vertex. **b** Tubule branch forming at three-way junction (rare tubule branches usually form at kinks) **c** filled cisterna, typical of triple mutant. **d** Right-hand tubule of polygon (**a**) dilates and becomes partially cisternal. **e** End of retraction cycle new branch from polygon. **f** End-on fusion of tubule branches. *Scale bar* = 4 μm

Branching and tubule extension can also be seen not only in latrunculin b-treated tissues (Hamada et al. 2014) but also in triple myosin XI insertional mutants (XI-K, XI-1 and XI-2; Fig. 2. These branches occur where the cortical ER network intersects the cortical microtubule network (Hamada et al. 2012; Hamada et al. 2014). These intersections are also sites where organelles pause in the cortex (Hamada et al. 2012); Golgi bodies, mitochondria and peroxisomes slow and then resume at normal speed. As described below, these pausing sites are regions (termed C-MERS for cortical microtubule ER sites) where viral replication complexes aggregate and form (Pena and Heinlein 2013). Once an ER branch is formed at these microtubule intersections and starts tracking along the microtubule (in latrunculin b-treated tissue), the movement is similar in rate and movement to some forms of microtubule tracking of the ER in animal cells (Wozniak et al. 2009). The remarkable movement of two distant ER tubules towards each other and subsequent end-on fusion shown in Fig. 2 could indicate that the tubules are tracking on some common element, such as a microtubule or microtubule bundle, in opposite directions. Indeed, when ER tubules appear to track along pre-existing microtubules, it can occur in both the (+) end and (−) end directions (Hamada et al. 2014). However, perhaps more important than microtubule-associated movement of the ER, which is slow and relatively rare, is the positive correlation with blunt ends and three-way junctions that are hypothesized to persist for long periods of time and therefore be sites of ER-PM MCSs. However, in order to quantify persistency, morphometric analyses, such as that done in persistency mapping, are needed (Sparkes et al. 2009a). As described below, during tip growth, internal ER tracking along endoplasmic microtubules are involved in generating an ER scaffold that provides a structure that is necessary for outward polarized growth but not for polarity initiation.

Actin-dependent movement implicates the actin-dependent motors: the myosins. As with many plant gene families, the myosin family is large and is comprised of two classes: XI and VIII. Arabidopsis, moss and rice encode for 17, 8 and 14 myosins respectively (Jiang and Ramachandran 2004; Muhlhausen and Kollmar 2013; Peremyslov et al. 2011; Reddy and Day 2001; Vidali et al. 2010). Class XI is similar to class V myosins due to a distinctive dilute domain in the carboxy terminus. Like members of the class V family, class XI members are involved in controlling organelle movement.

An in vitro technique, which reconstituted a network from a microsomal fraction that resembled the ER, indicated the involvement of a class XI myosin, actin and energy (ATP/GTP) (Yokota et al. 2011). The requirement for GTP is likely to be due to RHD3, a GTPase (see references in Stefano et al. 2014a).

Initial studies using complementary in vivo techniques, the analysis of T-DNA insertional mutants and expression of dominant negative myosins lacking the motor domain revealed that myosin XI-K (or Myo 11E, (Muhlhausen and Kollmar 2013)) is important for ER morphological remodelling (Griffing et al. 2014; Sparkes et al. 2009a; Ueda et al. 2010). Further studies using dominant negative tail domains of other Arabidopsis myosin XI paralogs revealed that XI-1 (Myo 11F), XI-C (Myo 11C1), XI-E (Myo 11C2) and XI-I (Myo 11G) also control morphological remodelling, but the effect on remodelling varies, XI-K and XI-1 having effects that differ from XI-C and E, see Table 1 and Griffing et al. (2014). It should, however, be noted that myosin XI-2 tail domain expression had very little effect on ER morphology, as determined by persistency mapping (Griffing et al. 2014), and the mutation had little effect on ER streaming or morphology alone (Ueda et al. 2010). However, when combined as a double mutant with the XI-K mutant, XI-2 mutation significantly changed ER streaming and altered the organization of actin in the cell (Ueda et al. 2010). In other triple mutants (XI-1, XI-2 and XI-K) actin organization has been shown to be perturbed, but the potential key role of myosin XI-2 could not be dissected out (Cai et al. 2014; Peremyslov et al. 2010). It should be noted that the effect of the myosin mutation on the ER, called ER streaming, was determined by Ueda et al. with an optic flow method, which combines changes in luminal flows with changes in the form of the ER. Since these two processes are different, and potentially unlinked, the differing result with myosin XI-1, for instance, which has little effect on ER streaming in the absence of other mutations (Ueda et al. 2010) could be the consequence of it having little effect on luminal flow. It should be noted that all of the myosin paralogs (XI-1, XI-K, XI-C, XI-E and XI-I) that affect ER morphology also control the movement of spheroid organelles, except myosin XI-2 which has no significant effect on ER morphology, but causes a large decrease in movement of mitochondria, Golgi and peroxisomes (Avisar et al. 2009; Avisar et al. 2008; Peremyslov et al. 2008; Peremyslov et al. 2010; Sparkes et al. 2008).

**Table 1:** Activity of actin and myosin tail domain expression (dominant negative) on ER form and dynamics in tobacco leaf epidermal cells (Griffing et al. 2014; Sparkes et al. 2009a)

Actin-myosin component	Events inhibited with drugs or tail domain expression	Effect on ER persistency
Actin polymerization and filaments	Tubule growth, active sliding of non-persistent nodes	Tubules neither grow or shrink—increased persistency, large cisternae develop and have increased persistency, increased persistency of entire network
Myosin XI-K (Myo 11E) and myosin XI-1 (Myo 11F)	Cisternal opening, final ring closure, network deformation and tubule shrinkage and growth	In the absence of cisternal opening, larger cisternae, larger mesh size, fewer meshes. In the absence of final ring closure, rings accumulate at junctions contributing to persistent cisternal size. With reduced tubule growth, tubules can make it to other tubules and cannot shrink leading to more blind-end persistent tubules.
Myosin XI-C (Myo 11C1) and myosin XI-E (Myo 11C2)	Tubules grow, but do not shrink, tubule sliding decreases, reduction in number of tubules to make new meshes.	Increased tubule persistency and less ring closure, creating more ‘default’ polygon filling and more, but smaller cisternae, mesh number reduced, size increases
Myosin XI-I (Myo 11G)	Absence of tubulation from existing cisternae	Larger persistent cisternae and fewer tubules. Larger cisternae decrease mesh number.
Myosin XI-2 (Myo 11B2)	Little change in form	

The maize gene, opaque 1, is involved in protein body formation and, reportedly, ER movement (Wang et al. 2012). This protein is, however, most closely related to the Arabidopsis myosin XI-I, which has been shown to localize to the nuclear envelope (Avisar et al. 2009; Tamura et al. 2013). It is apparently involved in the movement of the nucleus (Tamura et al. 2013) and is associated with the outer nuclear envelope via the outer envelope proteins: WIT1 and WIT2. Because it is also involved in tubulation of ER cisternae (Table 1), it may function in the tubulation of the nuclear envelope, a cisternal subdomain of the ER. Exciting future work on this motor protein may reveal if and how ER dynamics at the nuclear envelope influences nuclear motility.

Both XIK and XI-2 have been co-located to the ER in subcellular fractions. An antibody against a 175 kDa tobacco myosin labelled a fraction which co-sedimented with the ER and labelled ER-like structures in BY2 cells (Yokota et al. 2009). Since XI-2 has 75 % similarity to the 175 kDa tobacco myosin, XI-2 localization to the ER was inferred. Likewise, immunoblotting of sucrose density gradient fractions with an XIK peptide antibody inferred ER localization (Ueda et al. 2010). However, expression of a functional full-length myosin XI-K fluorescent fusion did not collocate to the ER (Park and Nebenfuhr 2013) and appeared to locate to unknown motile vesicles (Peremyslov et al. 2012). These apparent discrepancies in determining XI-K localization could be due to tissue-specific differences or may reflect a potential transient interaction either directly with the ER or through interaction with unknown vesicles. Here, XI-K may not associate with the entire network in vivo, and the regions it binds too may not be for prolonged periods of time. This type of behaviour would make it difficult to observe in real time and reconcile localization to the ER network. In addition, the presence of ER fragments in other membrane fractions due to MCSs (for example, plastid ER-enriched fractions, Andersson et al. 2007) compounds the issue of determining XI-K localization.

Potential functional redundancy and ‘interaction’ of one myosin with several organelles have confounded our understanding of the factors that specifically control the movement of a certain organelle class (Geitmann and Nebenfuhr 2015). Progress has been made with the identification of a myosin receptor family, although it is not clear if and how members of the family control organelle movement (Peremyslov et al. 2015; Peremyslov et al. 2013). Initial studies indicate interaction with myosins, co-location to unknown vesicles and effects on the dynamics of several organelles to which they do not co-locate. How can these results be reconciled? More specifically in the context of ER motility, how can several myosins control ER dynamics yet not co-locate to the organelle itself? It could be argued that co-location studies using truncated myosin fusions may not necessarily reflect the location of the native protein or that levels of association with the organelle are masked (obscured) by the cytoplasmic population. However, myosin XI-I, for example, was found to reside on the nucleus using both truncated and full-length fluorescent fusions (Avisar et al. 2009; Tamura et al. 2013). Hence, technical unknowns and limitations of experimental systems again raise the question, how can several myosins control the movement of the ER if they do not appear to co-locate to its surface? Could movement be driven by an alternative mechanism?

**Fig. 3:**
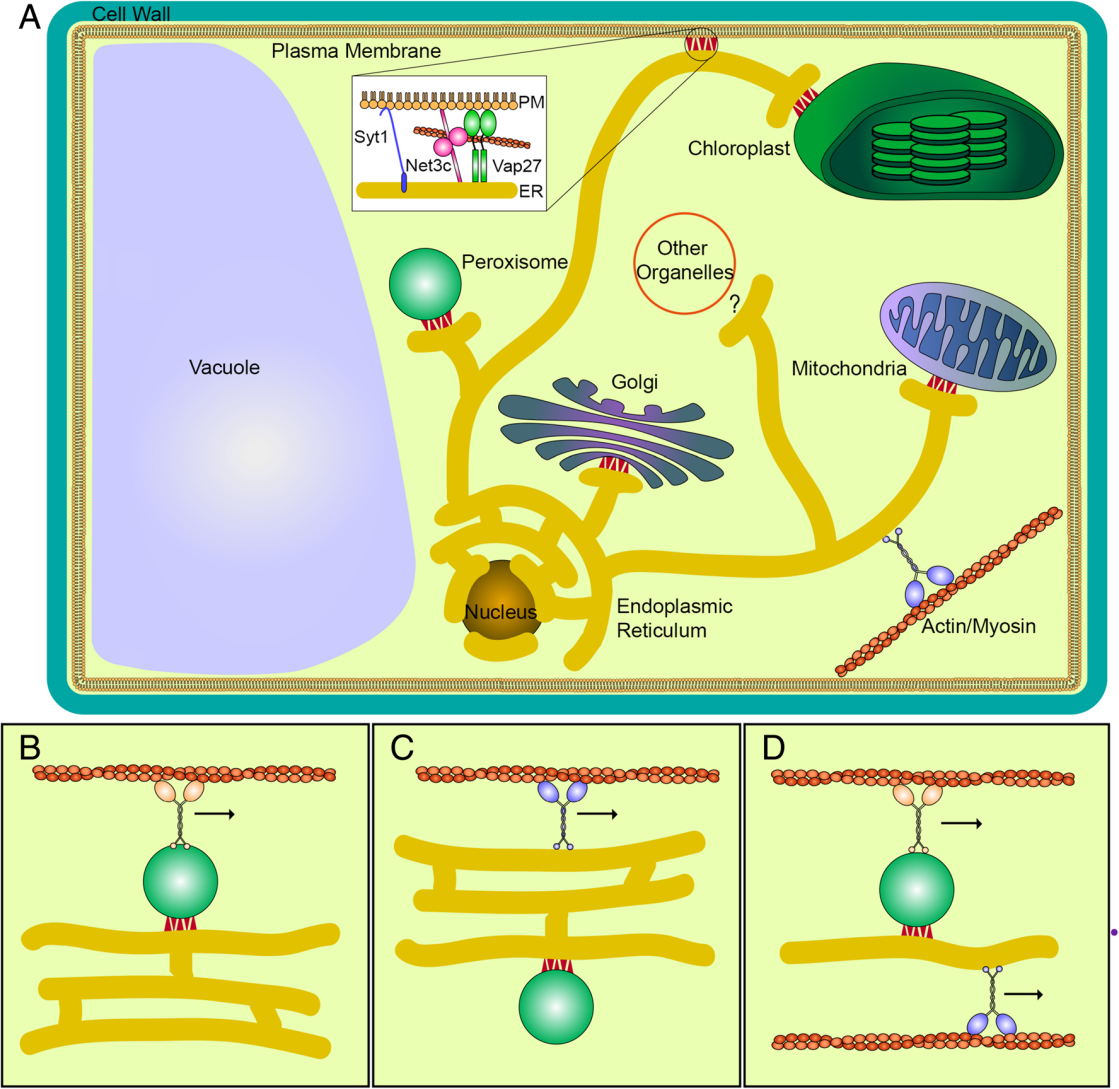
Schematic representation of molecular control of plant ER movement. Organelle interactions with the ER (*yellow*) are depicted where *red triangles* refer to reported (or inferred) interactions, and known molecular components for ER-PM are highlighted (**a**). Different models for how ER-organelle-driven motion may control ER movement (**b–d**). The ER might be pulled by a moving organelle tethered to the ER itself (**b**), the moving ER might carry the tethered organelle (**c**) or the movement of tethered organelles are driven through coordinated action of motors specific for each organelle (**d**)

Organelle movement is traditionally thought of as a cytoskeletal-dependent process with specificity being provided through direct motor association (usually in a heteroligomeric complex with recruitment factors) with a target organelle whose movement it controls. However, organelles, viewed as discrete membrane-bound compartments, can actually physically interact with other organelles (Fig. 3a). The tethering interaction between the two compartments could, in turn, control the movement where one organelle is bound by the motor, which, in turn, moves the target organelle and the organelle to which it is tethered too (Fig. 3b, c). Since there is a physical association between Golgi and the ER, it was hypothesized that Golgi motion, in turn, drove ER movement in plant cells (Sparkes et al. 2009b). However, BFA studies, which in effect causes Golgi resorption into the ER thereby removing Golgi stacks from the system, still allowed ER remodelling to occur (Sparkes et al. 2009a). ER morphology was affected and became more cisternal, but the network was still able to remodel. Therefore, were Golgi interaction to drive ER movement, it cannot be the sole driving factor, or alternatively Golgi stacks do not play a role in ER remodelling. Therefore, lack of a specific myosin interaction with the ER could potentially still drive movement by directly controlling the movement of an organelle which is tethered to the ER, which then indirectly affects the movement of the ER itself (Fig. 3b).

**Fig. 4:**
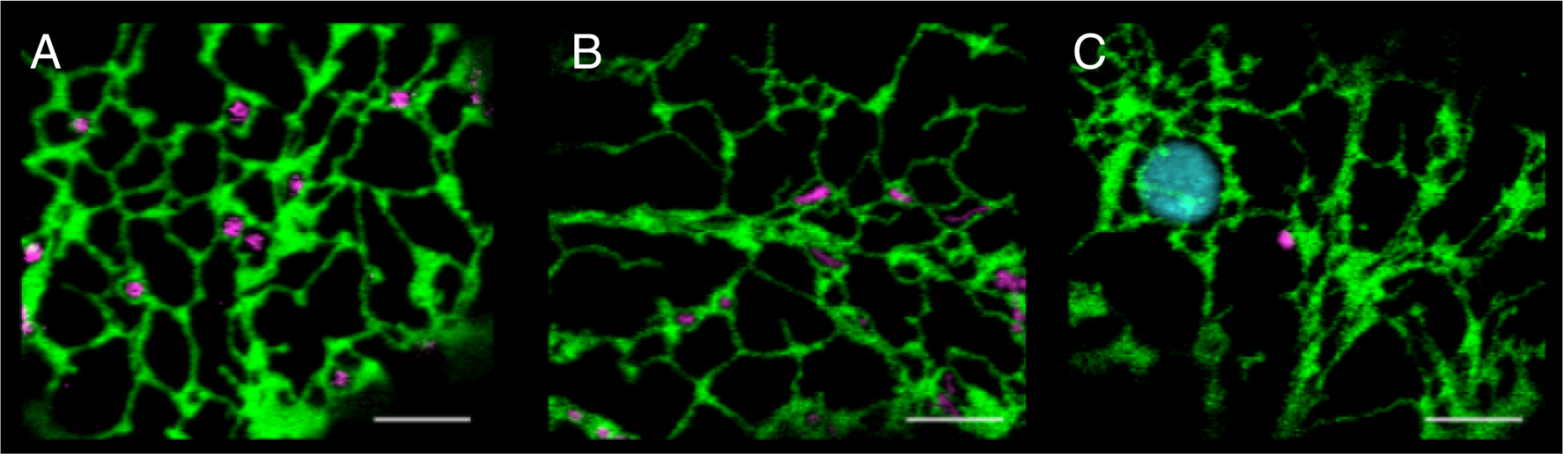
Close positioning of organelles next to the ER in tobacco leaf epidermal cells. Confocal images of tobacco leaf epidermal cells expressing an ER luminal marker shown in *green*, with organelle markers shown in *magenta*: **a** Golgi, **b** mitochondria and **c** peroxisomes. **c** Chloroplast autofluoresence is shown in *cyan*. Images taken from Supp. Movie 2. *Scale bar* = 5 μm

Additional studies have implicated ER-organelle tethering mechanisms with other highly motile organelles including peroxisomes and mitochondria (Jaipargas et al. 2015; Sinclair et al. 2009). Here, association is inferred from subcellular co-alignment between the organelles, which could potentially occur through close apposition of organelles in a highly constrained cortical cytoplasmic zone in highly vacuolated cells. Similarly, an interaction between the ER and chloroplasts or tubular chloroplast extensions (stromules) is based on co-alignment (Schattat et al. 2011). Figure 4 and Supp Movie 2 highlights the close association seen between the ER and Golgi, peroxisomes and mitochondria in highly vacuolated tobacco leaf epidermal cells. Additional evidence for chloroplast interactions with the ER comes from biochemical complementation and optical tweezer studies (Andersson et al. 2007; Mehrshahi et al. 2013). Biochemical complementation refers to synthetically targeting enzymes, which normally reside in either organelle A or B in wild-type cells, to the opposite organelle in a null background. Under such conditions, non-polar compounds synthesized in the chloroplast were accessible to the ER, indicating close association and shuttling of metabolites (Mehrshahi et al. 2013). Trapping chloroplasts using optical tweezers in laser ablated cells allowed the organelles to be pulled out of the lysed cell. Under such conditions, the ER appeared to be attached as it too was extended and extracted from the cell along with the trapped chloroplast (Andersson et al. 2007). The ER-chloroplast functional association is also revealed after brief photostimuation of the ER-chloroplast nexus, which results in a ‘shock wave’ of radiating ER movement and luminal/surface flow changes (Griffing 2011). The ER recovers normal movement and luminal flows within seconds after the photostimulation. Photostimulation of other regions of the ER or its nexus with other organelles does not produce this response. Interactions occurring between other organelles, such as peroxisome-chloroplast and chloroplast-nucleus, have also been documented, and it remains to be seen whether these interactions could in fact be mediated by an ER bridge between the two organelles (Gao et al. 2015; Higa et al. 2014).

Cytoplasmic streaming, which is fast bulk motion of the cytoplasm, occurs at a rapid rate with streams continually rotating and branching throughout cells undergoing radial growth. Cells undergoing polar tip growth have more ordered streaming patterns referred to as reverse fountain streaming, except in mosses (see below), where cytoplasmic streaming is not present. Often, spheroid organelles such as Golgi, peroxisome and mitochondria can be seen moving at fast rates within the stream, giving the impression that organelle motion is passive in these regions as they ‘slip’ into the fast stream. Intriguingly, Kachar et al. inferred that ER dynamics were linked to controlling cytoplasmic streaming (Kachar and Reese 1988). Likewise, modelling of streaming rates indicated that fluid motion in streams could not be due to movement of filaments (cytoskeletal components) or small spheroid structures (small organelles) and was likely driven by movement of a net-like structure (ER) within the cell generating enough shear force to, in turn, drag the cytoplasm to generate flow (Nothangel and Webb 1982). Subsequent observations of apparent coordinated organelle and ER motion lend some support to this model (Stefano et al. 2014b). However, again, it is difficult to reconcile whether the cytoplasmic streaming is due to ER motion or whether organelles tethered to the ER, which, in turn, move the ER, then affect streaming rates. This is an extremely complex problem. The basic model of motors specifically controlling the movement of one organelle (or even several organelles) does not therefore reflect the model system as a whole where organelle tethering and cytoplasmic flows also impact on organelle movement and positioning. ER movement is also likely controlled through interaction with the plasma membrane. Early observations of ER network dynamics led authors to hypothesize that there was a physical connection which may control and stabilize ER geometry (Lichtscheidl and Url 1990). More recent studies have been able to quantify these regions and determine the molecular components involved in the association with the plasma membrane (Levy et al. 2015; Perez-Sancho et al. 2015; Sparkes et al. 2009a; Wang et al. 2014).

## Cellular and organismal control of ER dynamics

### ER dynamics in tip growth

**Fig. 5:**
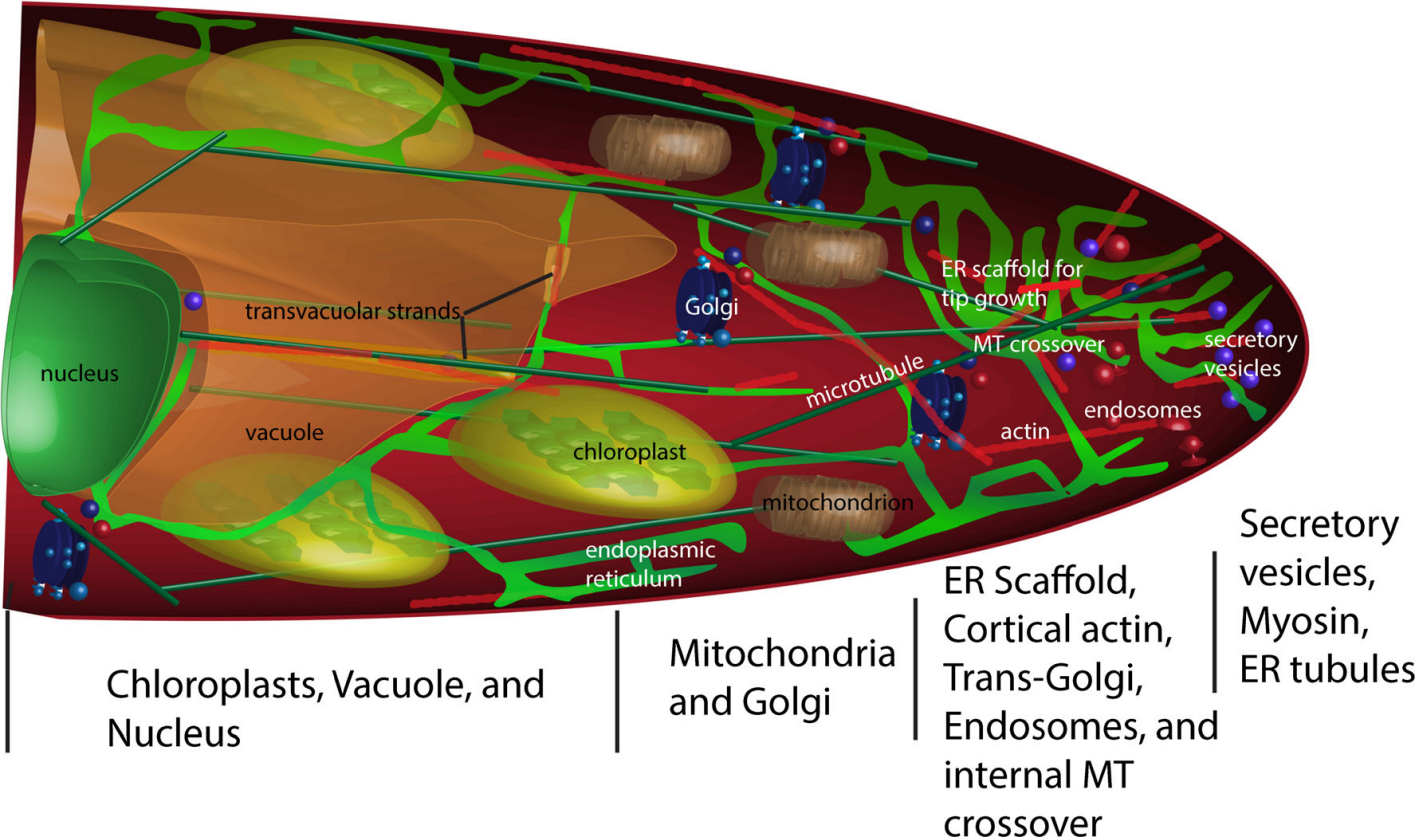
A schematic diagram of a tip-growing cell showing the polarized organization of organelles in relation to the endoplasmic reticulum. Although not shown, myosin is highest at the apical side of the ER scaffold, near the point of MT crossover. The scaffold is shown as a complex, condensed network of ER tubules, some of which emerge and transiently connect with the plasma membrane in the apex

Tip-growing plant cells show a highly polarized organization of organelles (see Fig. 5), with the ER, along with secretory and endocytic vesicles, being enriched in the so-called clear zone free of cytoplasmic organelles that occurs at the apex of tip-growing cells (Rounds and Bezanilla 2013). Mitochondria and Golgi stacks are enriched in a more distant but still subapical region (Furt et al. 2012). Behind these are found the peroxisomes, plastids, the vacuole and ultimately the nucleus (Fig. 5). Because exocytosis and endocytosis occur primarily at the apex of tip-growing cells, the presence of secretory and endocytic vesicles in the apex is easily explained (Ketelaar et al. 2008). In root hairs and pollen tubes approximately 87 and 79 %, respectively, more membrane is apically inserted by exocytosis than is needed to maintain the growing plasma membrane.

**Fig. 6:**
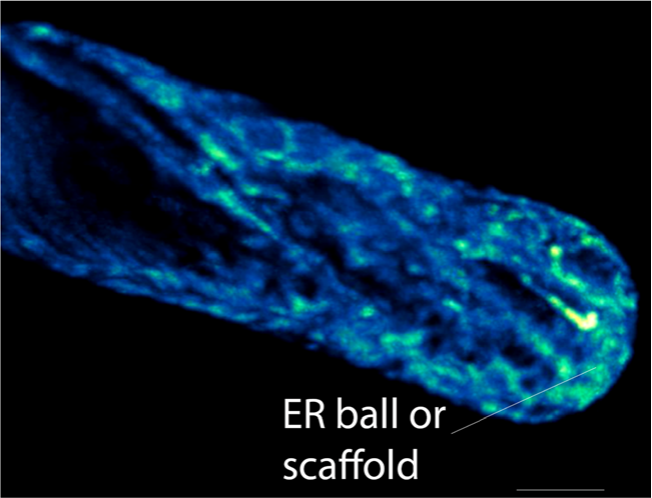
The presence of an ER ball or scaffold at the apex of a growing root hair of *Nicotiana benthamiana* constitutively expressing GFP-HDEL. Image is an average intensity projection of a series of optical sections taken with a lattice light sheet microscope in collaboration with John Heddleston and Teng-Leong Chew at the Advanced Imaging Center in Janelia-HHMI Research Campus, Ashburn VA. *Scale bar* = 5 μm

Less easily explained is the presence of the pervasive ER network and ER tubules at the tip. Evidence that this network is required for tip growth can be found in pollen tubes, where a lenticular subapical ‘platform’ of ER from which individual tubules emerge is required for fast growth (Lovy-Wheeler et al. 2007), and also in Characean algal rhizoids, where a complicated subapical ‘ball’ of aggregated ER is required for maximal tip growth (Braun 2001; Limbach et al. 2008). ER at the apex of tip-growing moss protonema has been observed by electron microscopy (McCauley and Hepler 1992). The ER at the tip of root hairs is so dynamic that its structure is best revealed with high-speed spinning disk confocal microscopy or high-speed lattice light sheet microscopy (Fig. 6) where a subapical ER aggregate is seen, along with tubules that invest the very tip (Perrine-Walker et al. 2014a). We propose that there are at least three important, not mutually exclusive, functions for ER in the clear zone: (1) It could interact through membrane contact sites with the endocytic sorting machinery which internalizes and recycles selected sterols, phospholipids, wall components or extracellular signals and their receptors, (2) it could maintain phospholipid and sterol balance and specialization in the apical plasma membrane through specific plasma membrane contact sites and (3) it could act as a plasma membrane-tethered scaffold which provides a structure for pushing the tip forward, being focused at the tip through potential associations with the crossover point of internal plus end-directed microtubules (Fig. 5). Because the terms ER ‘aggregate’ or platform are insufficient and potentially misleading (aggregation often being the result of aberrant self assembly processes and platforms being static), we have adopted the term ‘ER scaffold’ for this discussion because scaffolds are organized, but movable, assembly sites that are often attached to the object being assembled. Evidence for each of these possible functions of the ER scaffold will now be considered, starting with the last.

Evidence that the ER could act as a scaffold for building the tip includes work on brown algal zygote polarization, a classic model system for polar axis fixation (Quatrano et al. 1985). By 4 h after fertilization of the alga *Silvetia compressa*, a polar axis is formed on the dark side of a light vector and manifests itself by local secretion of adhesive at the prospective rhizoid tip, along with an increased number of microtubules aligned along the polar axis, emanating from the central nucleus and terminating at the prospective rhizoid tip (Peters and Kropf 2010). After a further 2 h (6 h after fertilization), the ER is preferentially co-localized with the increased microtubules along the polar axis. In zygotes treated with oryzalin 30 min after fertilization, the microtubules depolymerize and, although there is the formation of a rhizoidal region by 24 h, the rhizoid does not grow outward as it does in control, untreated zygotes. In control zygotes, the cytoplasmic microtubules in the thallus side of the 24 h embryo have little or no associated ER, whereas the growing rhizoid pole has abundant subapical ER associated with cytoplasmic microtubules. These experiments support a model whereby tip growth outward from the site of polarization is controlled by the ER-microtubule (ER-MT) scaffold, driven forward by the enlarging, turgor-driven vacuole.

The proposed nature of the ER scaffold is that it is organized as a complex of tubules that cluster through interaction with the cytoskeleton and selective tethering to the plasma membrane. Tethering to the plasma membrane would arise through MCSs (‘anchor sites’, as described in Sparkes et al. 2009a, b) which would be enriched for ER-localized proteins involved in lipid transport and close association between the membranes (Gatta et al. 2015; Schauder et al. 2014). The specific enrichment of sterols in the apical region of the plasma membrane of growing root hairs (Grebe et al. 2003; Ovecka et al. 2010) would suggest that there is a special form of sterol transport that occurs near the tip of the growing cell and special tethers may perform this role. Furthermore, many of the enzymes involved in sterol metabolism are found in both the ER and PM (Silvestro et al. 2013). Other forms of lipid transfer at the ER-PM MCS may occur, including the control of inositol phosphates and phosphatidylserine (Chung et al. 2015). In this regard, it is particularly interesting to note that pollen tubes take up the fluorescently modified phospholipid, bis-BODIPY phosphocholine, not into endosomal vesicles but into the ER in a region just behind the apex (Lisboa et al. 2008), where the platform of ER (Lovy-Wheeler et al. 2007) resides. This internalization appears to be into the internal, not the cortical, ER based on its internal, but not cortical, co-localization with DiOC6. The ER localization of this fluorescent phospholipid in pollen tubes is very similar to the putative ER localization of the enzyme involved in the conversion of 2,4 methylene cholesterol in plants (Klahre et al. 1998), which has been confirmed in other cell types (Silvestro et al. 2013).

Several functional associations between the ER and endosomes have been recently reported in animal and fungal cells, including involvement in intracellular calcium and sterol regulation (van der Kant and Neefjes 2014), endosomal maturation (Friedman et al. 2013) and fission of endosomes (Rowland et al. 2014). Of particular interest, in relation to tip growth, is the finding that neurite outgrowth is mediated by the association of endosomes with the ER, an association which facilitates the transfer of the vesicles to microtubules for kinesin-based movement to the tip where they fuse (Raiborg et al. 2015). The association of endocytic vesicles of various kinds with the ER scaffold in Chara is consistent with a similar function (Limbach et al. 2008), although the nature of the exact connection between these vesicles and the ER scaffold is difficult to analyze because in the high-pressure frozen, freeze-substituted rhizoids, ER was difficult to visualize. In fact, modulation of vesicle tracking (endocytic, recycling or exocytic) at the ER scaffold may be one of its additional functions. The coordination of traffic between the microtubule cytoskeleton and the actin cytoskeleton in tip growth has been a major conundrum, but the ER scaffold may play a role here. The tip of the ER scaffold may be the site for the accumulation of myosin XI-K at the tips of root hairs (Park and Nebenfuhr 2013) but may not be responsible for the formation and maintenance of the ER scaffold, since the ER network is not greatly changed in mutants in XI-K. The tip of the ER scaffold is the site for myosin accumulation in the tips of growing caulonema in moss (Furt et al. 2013). Several groups have shown the same or similar localization of a crossover point for cytoplasmic microtubules in root hairs (Lloyd et al. 1987; Perrine-Walker et al. 2014b; Sieberer et al. 2005; Van Bruaene et al. 2004). However, in Chara rhizoids, longitudinal microtubules stop well before the ER scaffold, whereas actin is found directly at its apex (Braun and Wasteneys 1998). In the tips of moss caulonema, the kinesin (+)-end-directed motors KINID1a and KINID1b and the end-binding microtubule protein EB1 are found at the point of crossover (Hiwatashi et al. 2014). Future experiments will reveal if the ER scaffold participates in the transfer of vesicle traffic from one kind of cytoskeletal element to the other—it is certainly in the right place!

### Relocalization of ER in response to biotic signals

The following section covers changes in ER dynamics in response to several biotic stimuli including bacteria, arbuscular mycorrhizas, fungi (including *Peronospora parasitica* and *Blumeria graminis f*. sp. *hordei*) and viral infection. It remains to be seen whether the mechanisms that drive ER responses under differing biotic stresses are conserved as comprehensive studies on the role of actin and microtubules and how they intersect and drive ER dynamics are incomplete. Similarly, it is not clear whether responses are driven by the host, pathogen or a combination of both during the defence response.

The association of bacteria with symbiotic root hairs is an excellent place to start considering how the ER changes in response to association with symbiotic bacteria, such as *Mesorhizobium loti* (Perrine-Walker et al. 2014a). In the growing root hair, the ‘condensed ER form’ described above as the ER scaffold persists in the tip in actively growing hairs, but once growth has stopped, the ER becomes an ‘open reticulum’ (Perrine-Walker et al. 2014a). However, after Nod factor or *M. loti* exposure, the ER scaffold continues to persist in the tip as the hair curls. If the hair contains a colony of bacteria in the bend of the curl, the ER continues to show an ER scaffold in the bend and as the infection thread is initiated and grows towards the nucleus, it is surrounded by the scaffold or the ‘condensed’ ER. Specific cell wall factors in the *Medicago truncatula*-*Sinorhizobium meliloti* symbiosis are initially laid down (MtENOD11) in the curved region where the bacterium resides, accompanied by an increase in local exocytosis as monitored with MtVAMP721, which is interpreted to be the first stage, the formation of the infection chamber in the bend of the root, of a two stage process of infection thread formation (Fournier et al. 2015). We propose, based on the work of the Ridge group (Perrine-Walker et al. 2014a), that the further establishment of the infection thread, the second stage, may also be under direct control from an ER scaffold. Instead of being constructed by wall secretion, the association of the infection thread with the ER MCS could provide the lipid required for internal infection thread membrane. The microtubule dynamics associated with these processes are consistent with the ER scaffold being under endoplasmic microtubule control. Endoplasmic microtubules form crossover clusters in the region where the ER scaffold lies in the bend in the root hair during infection (Perrine-Walker et al. 2014b). Furthermore, as the infection thread develops, the endoplasmic microtubules track along it longitudinally.

A similar ER-MT reorganization occurs upon infection of root epidermal cells of *Medicago trucatula* with the arbuscular mycorrhiza *Gigaspora gigantea* or *Gigaspora rosea* (Genre et al. 2005). At the appressorium contact site after infection with *Gigaspora*, the ER undergoes marked changes from an open reticulum into a condensed form similar to the ER scaffold found in tip growth. Accompanying this change, the MT and actin filaments reorganize at the appressorium contact site. In this case, however, the scaffold appears to directly include the nucleus (and nuclear envelope) at the early stages, which moves towards the appressorium contact site within 2 h of infection. The microtubules lose their primarily transverse orientation and generate crossover points near the region of fungal penetration. The inclusion of the nucleus in the formation of this ‘pre-penetration apparatus’ is not dissimilar to the situation in root hairs or moss protonema where the nucleus trails at a specific distance behind the growing tip (Lloyd et al. 1987) and the polymerization of microtubules at the nuclear envelope contributes to the endoplasmic microtubule population (Braun and Wasteneys 1998). Interestingly, the aggregated ER-nuclear envelope structure occurs along the same axis in adjacent cells, appearing as polarized ER structures along files of cortical cells which will participate in forming the pre-penetration apparatus (Genre et al. 2008).

In pioneering studies by the Hardham lab, it was discovered that the actin network and the ER network reorganized at the site of *P. parasitica* and *B. graminis f.* sp. *hordei* (powdery mildew) infection in Arabidopsis leaves (Takemoto et al. 2003; Takemoto et al. 2006). The reorganization also included accumulation at the infection site of other organelles, such as Golgi stacks. In contrast to the situation with symbiotic associations, the reorganization did not include the microtubule network. The microtubule network, while not reorganizing, may be involved since microtubule-associated ROP-GTPase activating protein (MAGAP1) is locally mobilized to decorate cortical microtubules upon infection (Hoefle et al. 2011). Recent studies show that the reorganization of the actin network and the aggregation of mitochondria, endosomes and Golgi (ER was not determined) upon fungal penetration did not occur as readily in myosin XI mutants (Yang et al. 2014). The reorganization is not, however, strictly dependent on fungal infection. In an elegant demonstration of the abiotic component of this response, simply pressing on epidermal cells with a microneedle caused reorganization of the actin network and the ER, causing it to swirl around the pressure point and concentrate upon it (Hardham et al. 2008). Although the molecular mechanism of this response is unknown, it appears that it is not so much one of growth as it is reorganization. For example, an ATP-binding cassette (ABC) transporter protein, PEN3, is recruited to the site of fungal penetration but not by vesicular means (Underwood and Somerville 2013). Non-vesicular transport of this nature might involve reorganized ER-PM tethering or transport.

The ER undergoes massive reorganization upon some forms of viral infection, such as tobacco mosaic virus where the viral movement protein is targeted to plasmodesmata via the ER-actin network (Wright et al. 2007; Reichel and Beachy 1998). The central ER desmotubule and the plasma membrane of plasmodesmata (PD) form a particular kind of ER-PM MCS, and several potential candidate proteins involved in such attachment have been identified by interaction with a reticulon found in plasmodesmata (Knox et al. 2015; Kriechbaumer et al. 2015). The space can be modified by viral infection or viral movement protein, as can the local ER-PM attachment adjacent to the plasmodesmata, recruiting a synaptotagmin typical of ER-PM associations, SYTA (Levy et al. 2015) to plasmodesmata sites, and thereby changing the ability of the PD to transport virus. SYTA is reported to be a plasma membrane protein (Kriechbaumer et al. 2015; Uchiyama et al. 2014), while other extended synaptotagmins in animal and yeast cells (called tricalbins in yeast) are ER-localized. Although tobamovirus viral replication complexes (VRCs) contain SYTA at the plasmodesmatal site, not all viruses that are subject to cell-to-cell transport regulation by SYTA have VRCs associated with plasmodesmata (Uchiyama et al. 2014). The potential for ER-endosome interaction exists, since SYTA has been reported to regulate endocytosis as well (Lewis and Lazarowitz 2010). The same synaptotagmin has been shown to be relatively immobile in the plasma membrane (Perez-Sancho et al. 2015), so how movement protein recruits it to plasmodesmatal sites is unclear but could involve endocytosis.

The relationship between some VRCs and the ER is clear: The VRCs move along the ER network, pausing at regions where there are microtubule intersections (Pena and Heinlein 2013). Other organelles show similar pausing at microtubule intersections with the ER (Hamada et al. 2012), and it is at these junctions where the ER intersects the microtubules in the cell cortex and may branch and track upon the microtubules (Hamada et al. 2014). These ER-MT intersections are fairly immobile, except, of course, when the ER tubule is tracking along the microtubule. It has been proposed that these C-MERS are involved in the establishment and transport of VRCs, as well as cellulose synthase deposition, endosome trafficking of plasma membrane PIN 2 proteins and transport of non-cell-autonomous transcription factors (Pena and Heinlein 2013). However, absent from these models is the potential involvement of ER-PM MCSs. Indeed, when the relationship between microtubules and ER-PM MCSs are analyzed, the MCSs are adjacent to microtubules and not superimposed on the microtubules (Perez-Sancho et al. 2015). As discussed above for tip growth, the tethering of the ER to the plasma membrane may be independent from its association with the microtubule network, but both may work together to generate a functional ER complex. In tip growth, that complex is the ER scaffold, while in viral infection, that complex is the VRC.

## Concluding remarks

Here, we have given an overview of the components that drive ER network dynamics, their potential communication with other organelles, and provide a model as to how these may play a role during development and in response to stresses. The role of the ER as a connecting element, through membrane contact sites, with most, if not all, of the internal spheroid organelles is now clear, if not in plants, then in other systems. These interactions may be transient in nature. In plants, the internal dynamics of rapidly directed transport of cellular organelles and cytoplasmic streaming exaggerate the changes in the ER and make the nature of the movement, in the context of these connections, even more interesting and complex to understand. Future developments of analytical platforms will allow the complex network to be unravelled and will enable specific roles of molecular components in producing this extremely important network to be identified.

## Electronic supplementary material

Below is the link to the electronic supplementary material.

ESM 1 Supp movie 1 The cortical ER in plant cells is highly dynamic. Confocal movie taken at 2 frames per second (s) of tobacco leaf epidermal cells expressing an ER luminal marker (GFP-HDEL) shown in green. Frames 0–60 (0–30 s) are displayed. Scale bar = 5 μm. (MOV 43.2 mb)

ESM 2 Supp movie 2 Close positioning of organelles next to the ER in tobacco leaf epidermal cells. Confocal movies (taken at 2 frames per second (s)) of tobacco leaf epidermal cells expressing an ER luminal marker (GFP-HDEL) shown in green, with organelle markers shown in magenta: (A) Golgi, (B) mitochondria and (C) peroxisomes. (C) Chloroplast autofluoresence is shown in cyan. Frames 0–60 (0–30 s) are displayed. Scale bar = 5 μm. (MOV 34.6 mb)

## References

[CR1] Andersson MX, Goksor M, Sandelius AS (2007). Optical manipulation reveals strong attracting forces at membane contact sites between endoplasmic reticulum and chloroplasts. The Journal of Biological Chemistry.

[CR2] Avisar D, Abu-Abied M, Belausov E, Sadot E, Hawes C, Sparkes IA (2009). A comparative study of the involvement of 17 Arabidopsis myosin family members on the motility of Golgi and other organelles. Plant Physiology.

[CR3] Avisar D, Prokhnevsky AI, Makarova KS, Koonin EV, Dolja VV (2008). Myosin XI-K is required for rapid trafficking of Golgi stacks, peroxisomes and mitochondria in leaf cells of Nicotiana benthamiana. Plant Physiology.

[CR4] Braun M (2001). Association of spectrin-like proteins with the actin-organized aggregate of endoplasmic reticulum in the Spitzenkorper of gravitropically tip-growing plant cells. Plant Physiol.

[CR5] Braun M, Wasteneys GO (1998). Distribution and dynamics of the cytoskeleton in graviresponding protonemata and rhizoids of characean algae: exclusion of microtubules and a convergence of actin filaments in the apex suggest an actin-mediated gravitropism. Planta.

[CR6] Cai C, Henty-Ridilla JL, Szymanski DB, Staiger CJ (2014). Arabidopsis myosin XI: a motor rules the tracks. Plant Physiol.

[CR7] Chung J (2015). INTRACELLULAR TRANSPORT. PI4P/phosphatidylserine countertransport at ORP5- and ORP8-mediated ER-plasma membrane contacts. Science.

[CR8] Foissner I, Menzel D, Wasteneys GO (2009). Microtubule-dependent motility and orientation of the cortical endoplasmic reticulum in elongating characean internodal cells. Cell motility and the cytoskeleton.

[CR9] Fournier J (2015). Remodeling of the infection chamber before infection thread formation reveals a two-step mechanism for rhizobial entry into the host legume root hair. Plant Physiol.

[CR10] Friedman JR, Dibenedetto JR, West M, Rowland AA, Voeltz GK (2013). Endoplasmic reticulum-endosome contact increases as endosomes traffic and mature. Mol Biol Cell.

[CR11] Furt F, Lemoi K, Tuzel E, Vidali L (2012). Quantitative analysis of organelle distribution and dynamics in Physcomitrella patens protonemal cells. BMC plant biology.

[CR12] Furt F, Liu YC, Bibeau JP, Tuzel E, Vidali L (2013). Apical myosin XI anticipates F-actin during polarized growth of Physcomitrella patens cells. Plant J.

[CR13] Gao H et al. (2015) In vivo quantification of peroxisome tethering to chloroplasts in tobacco epidermal cells using optical tweezers .Plant Physiol doi:10.1104/pp.15.01529 10.1104/pp.15.01529 PMC4704594 26518344

[CR14] Gatta AT, Wong LH, Sere YY, Calderon-Norena DM, Cockcroft S, Menon AK, Levine TP (2015) A new family of StART domain proteins at membrane contact sites has a role in ER-PM sterol transport eLife 4 doi:10.7554/eLife.07253 10.7554/eLife.07253 PMC4463742 26001273

[CR15] Geitmann A, Nebenfuhr A (2015). Navigating the plant cell: intracellular transport logistics in the green kingdom. Molecular Biology of the Cell.

[CR16] Genre A, Chabaud M, Faccio A, Barker DG, Bonfante P (2008). Prepenetration apparatus assembly precedes and predicts the colonization patterns of arbuscular mycorrhizal fungi within the root cortex of both Medicago truncatula and Daucus carota. Plant Cell.

[CR17] Genre A, Chabaud M, Timmers T, Bonfante P, Barker DG (2005). Arbuscular mycorrhizal fungi elicit a novel intracellular apparatus in Medicago truncatula root epidermal cells before infection. Plant Cell.

[CR18] Grebe M (2003). Arabidopsis sterol endocytosis involves actin-mediated trafficking via ARA6-positive early endosomes. Current biology : CB.

[CR19] Griffing LR (2010). Networking in the endoplasmic reticulum. Biochemical Society transactions.

[CR20] Griffing LR (2011). Laser stimulation of the chloroplast/endoplasmic reticulum nexus in tobacco transiently produces protein aggregates (boluses) within the endoplasmic reticulum and stimulates local ER remodeling. Molecular plant.

[CR21] Griffing LR, Gao HT, Sparkes I (2014) ER network dynamics are differentially controlled by myosins XI-K, XI-C, XI-E, XI-I, XI-1, and XI-2 Frontiers in plant science 5:218 doi:10.3389/fpls.2014.00218 10.3389/fpls.2014.00218 PMC4033215 24904614

[CR22] Hamada T (2012). RNA processing bodies, peroxisomes, Golgi bodies, mitochondria, and endoplasmic reticulum tubule junctions frequently pause at cortical microtubules. Plant & cell physiology.

[CR23] Hamada T, Ueda H, Kawase T, Hara-Nishimura I (2014). Microtubules contribute to tubule elongation and anchoring of endoplasmic reticulum, resulting in high network complexity in Arabidopsis. Plant Physiol.

[CR24] Hardham AR, Takemoto D, White RG (2008). Rapid and dynamic subcellular reorganization following mechanical stimulation of Arabidopsis epidermal cells mimics responses to fungal and oomycete attack. BMC Plant Biol.

[CR25] Higa T, Suetsugu N, Kong SG, Wada M (2014). Actin-dependent plastid movement is required for motive force generation in directional nuclear movement in plants. Proc Natl Acad Sci U S A.

[CR26] Hiwatashi Y, Sato Y, Doonan JH (2014). Kinesins have a dual function in organizing microtubules during both tip growth and cytokinesis in Physcomitrella patens. Plant Cell.

[CR27] Hoefle C, Huesmann C, Schultheiss H, Bornke F, Hensel G, Kumlehn J, Huckelhoven R (2011). A barley ROP GTPase ACTIVATING PROTEIN associates with microtubules and regulates entry of the barley powdery mildew fungus into leaf epidermal cells. Plant Cell.

[CR28] Jaipargas EA, Barton KA, Mathur N, Mathur J (2015) Mitochondrial pleomorphy in plant cells is driven by contiguous ER dynamics Frontiers in plant science 6:783 doi:10.3389/fpls.2015.00783 10.3389/fpls.2015.00783 PMC4585081 26442089

[CR29] Jiang SY, Ramachandran S (2004). Identification and molecular characterization of myosin gene family in Oryza sativa genome. Plant and Cell Physiology.

[CR30] Kachar B, Reese TS (1988). The mechanism of cytoplasmic streaming in characean algal cells: sliding of endoplasmic reticulum along actin filaments. Journal of Cell Biology.

[CR31] Ketelaar T, Galway ME, Mulder BM, Emons AM (2008). Rates of exocytosis and endocytosis in Arabidopsis root hairs and pollen tubes. J Microsc.

[CR32] Klahre U (1998). The Arabidopsis DIMINUTO/DWARF1 gene encodes a protein involved in steroid synthesis. Plant Cell.

[CR33] Knebel WH, Quader H, Schnepf E (1990). Mobile and immobile endoplasmic reticulum in onion bulb epidermis cells: short- and long-term observations with a confocal laser scanning microscope. European Journal of Cell Biology.

[CR34] Knox K (2015). Putting the Squeeze on Plasmodesmata: A Role for Reticulons in Primary Plasmodesmata Formation. Plant Physiol.

[CR35] Kriechbaumer V, Botchway SW, Slade SE, Knox K, Frigerio L, Oparka K, Hawes C (2015). Reticulomics: protein-protein interaction studies with two plasmodesmata-localized reticulon family proteins identify binding partners enriched at plasmodesmata, endoplasmic reticulum, and the plasma membrane. Plant Physiol.

[CR36] Lee HY (2012). Arabidopsis RTNLB1 and RTNLB2 Reticulon-like proteins regulate intracellular trafficking and activity of the FLS2 immune receptor. The Plant Cell.

[CR37] Lemarchand L, Euler R, Lin C, Sparkes I, Dediu AH, Martin-Vide C, Truthe B (2014). Modeling the geometry of the endoplasmic reticulum network. Algortihms for computational biology.

[CR38] Levy A, Zheng JY, Lazarowitz SG (2015). Synaptotagmin SYTA forms ER-plasma membrane junctions that are recruited to plasmodesmata for plant virus movement. Current biology : CB.

[CR39] Lewis JD, Lazarowitz SG (2010). Arabidopsis synaptotagmin SYTA regulates endocytosis and virus movement protein cell-to-cell transport. Proc Natl Acad Sci U S A.

[CR40] Lichtscheidl I, Url WG (1987). Investigation of the protoplasm of Allium cepa inner epidermal cells using ultraviolet microscopy. European Journal of Cell Biology.

[CR41] Lichtscheidl IK, Url WG (1990). Organisation and dynamics of cortical endoplasmic reticulum in inner epidermal cells of onion bulb scales. Protoplasma.

[CR42] Liebe S, Menzel D (1995). Actomyosin-based motility of endoplasmic reticulum and chloroplasts in Vallisneria mesophyll cells. Biology of the Cell.

[CR43] Limbach C, Staehelin LA, Sievers A, Braun M (2008). Electron tomographic characterization of a vacuolar reticulum and of six vesicle types that occupy different cytoplasmic domains in the apex of tip-growing. Chara rhizoids Planta.

[CR44] Lin C, Lemarchand L, Euler R, Sparkes I (2015) Modeling the geometry and dynamics of the Endoplasmic Reticulum network IEEE/ACM Transactions on Computational Biology and Bioinformatics 1 doi:doi:10.1109/TCBB.2015.2389226 10.1109/TCBB.2015.2389226 29610097

[CR45] Lin C, Zhang Y, Sparkes I, Ashwin P (2014). Structure and dynamics of ER: minimal networks and biophysical constraints. Biophysical Journal.

[CR46] Lisboa S, Scherer GE, Quader H (2008). Localized endocytosis in tobacco pollen tubes: visualisation and dynamics of membrane retrieval by a fluorescent phospholipid. Plant Cell Rep.

[CR47] Lloyd CW, Pearce KJ, Rawlins DJ, Ridge RW, Shaw PJ (1987). Endoplasmic microtubules connect the advancing nucleus to the tip of legume root hairs, but F-actin is involved in basipetal migration. Cell motility and the cytoskeleton.

[CR48] Lovy-Wheeler A, Cardenas L, Kunkel JG, Hepler PK (2007). Differential organelle movement on the actin cytoskeleton in lily pollen tubes. Cell motility and the cytoskeleton.

[CR49] McCauley MM, Hepler PK (1992). Cortical ultrastructure of freeze-substituted protonemata of the moss. Funaria hygrometrica Protoplasma.

[CR50] Mehrshahi P, Stefano G, Andaloro JM, Brandizzi F, Froehlich JE, DellaPenna D (2013). Transorganellar complementation redefines the biochemical continuity of endoplasmic reticulum and chloroplasts. Proc Natl Acad Sci U S A.

[CR51] Muhlhausen S, Kollmar M (2013). Whole genome duplication events in plant evolution reconstructed and predicted using myosin motor proteins. BMC Evol Biol.

[CR52] Nothangel EA, Webb WW (1982). Hydrodynamic models of viscous coupling between motile myosin and endoplasm in Characean algae. Journal of Cell Biology.

[CR53] Ovecka M, Berson T, Beck M, Derksen J, Samaj J, Baluska F, Lichtscheidl IK (2010). Structural sterols are involved in both the initiation and tip growth of root hairs in Arabidopsis thaliana. Plant Cell.

[CR54] Park E, Nebenfuhr A (2013) Myosin XIK of Arabidopsis thaliana accumulates at the root hair tip and is required for fast root hair growth .PloS one 8:e76745 doi:10.1371/journal.pone.0076745 10.1371/journal.pone.0076745 PMC3792037 24116145

[CR55] Pena EJ, Heinlein M (2013). Cortical microtubule-associated ER sites: organization centers of cell polarity and communication. Curr Opin Plant Biol.

[CR56] Peremyslov VV, Cole RA, Fowler JE, Dolja VV (2015). Myosin-powered membrane compartment drives cytoplasmic streaming, cell expansion and plant development. PLoS One.

[CR57] Peremyslov VV, Klocko AL, Fowler JE, Dolja VV (2012) Arabidopsis myosin XI-K localizes to the motile endomembrane vesicles associated with F-actin Frontiers in plant science 3:184 doi:10.3389/fpls.2012.00184 10.3389/fpls.2012.00184 PMC3432474 22969781

[CR58] Peremyslov VV (2011). Expression, splicing, and evolution of the myosin gene family in plants. Plant Physiology.

[CR59] Peremyslov VV, Morgun EA, Kurth EG, Makarova KS, Koonin EV, Dolja VV (2013). Identification of myosin XI receptors in Arabidopsis defines a distinct class of transport vesicles. Plant Cell.

[CR60] Peremyslov VV, Prokhnevsky AI, Avisar D, Dolja VV (2008). Two class XI myosins function in organelle trafficking and root hair development in Arabidopsis thaliana. Plant Physiology.

[CR61] Peremyslov VV, Prokhnevsky AI, Dolja VV (2010) Class XI myosins are required for development, cell expansion, and F-actin organization in Arabidopsis Plant Cell 22:1883–1897 doi:10.1105/tpc.110.076315 10.1105/tpc.110.076315 PMC2910955 20581304

[CR62] Perez-Sancho J et al. (2015) The Arabidopsis synaptotagmin1 is enriched in endoplasmic reticulum-plasma membrane contact sites and confers cellular resistance to mechanical stresses Plant Physiol 168:132–143 doi:10.1104/pp.15.00260 10.1104/pp.15.00260 PMC4424031 25792253

[CR63] Perrine-Walker FM, Kouchi H, Ridge RW (2014). Endoplasmic reticulum-targeted GFP reveals ER remodeling in Mesorhizobium-treated Lotus japonicus root hairs during root hair curling and infection thread formation. Protoplasma.

[CR64] Perrine-Walker FM, Lartaud M, Kouchi H, Ridge RW (2014). Microtubule array formation during root hair infection thread initiation and elongation in the Mesorhizobium-Lotus symbiosis. Protoplasma.

[CR65] Peters NT, Kropf DL (2010). Asymmetric microtubule arrays organize the endoplasmic reticulum during polarity establishment in the brown alga Silvetia compressa. Cytoskeleton.

[CR66] Quader H, Schnepf E (1986). Endoplasmic reticulum and cytoplasmic streaming: fluorescence microscopical observation in adaxial epidermis cells of onion bulb scales. Protoplasma.

[CR67] Quatrano RS, Griffing LR, Huber-Walchli V, Doubet RS (1985). Cytological and biochemical requirements for the establishment of a polar cell. Journal of cell science Supplement.

[CR68] Raiborg C, Wenzel EM, Stenmark H (2015) ER-endosome contact sites: molecular compositions and functions. EMBO J 34:1848–1858 doi:10.15252/embj.201591481 10.15252/embj.201591481 PMC4547891 26041457

[CR69] Reddy ASN, Day IS (2001). Analysis of the myosins encoded in the recently completed Arabidopsis thaliana genome sequence. Genome Biology.

[CR70] Reichel C, Beachy RN (1998). Tobacco mosaic virus infection induces severe morphological changes of the endoplasmic reticulum. Proc Natl Acad Sci U S A.

[CR71] Ridge RW, Uozumi Y, Plazinski J, Hurley UA, Williamson RE (1999). Developmental transitions and dynamics of the cortical ER of Arabidopsis cells seen with green fluorescent protein. Plant Cell Physiol.

[CR72] Rounds CM, Bezanilla M (2013). Growth mechanisms in tip-growing plant cells. Annu Rev Plant Biol.

[CR73] Rowland AA, Chitwood PJ, Phillips MJ, Voeltz GK (2014). ER contact sites define the position and timing of endosome fission. Cell.

[CR74] Runions J, Brach T, Kuhner S, Hawes C (2006). Photoactivation of GFP reveals protein dynamics within the endoplasmic reticulum membrane. J Exp Bot.

[CR75] Schattat M, Barton K, Baudisch B, Klosgen RB, Mathur J (2011). Plastid stromule branching coincides with contiguous endoplasmic reticulum dynamics. Plant Physiology.

[CR76] Schauder CM (2014). Structure of a lipid-bound extended synaptotagmin indicates a role in lipid transfer. Nature.

[CR77] Schweitzer Y, Shemesh T, Kozlov MM (2015). A model for shaping membrane sheets by protein scaffolds. Biophys J.

[CR78] Shemesh T (2014). A model for the generation and interconversion of ER morphologies. Proc Natl Acad Sci U S A.

[CR79] Sieberer BJ, Ketelaar T, Esseling JJ, Emons AM (2005). Microtubules guide root hair tip growth. The New phytologist.

[CR80] Silvestro D, Andersen TG, Schaller H, Jensen PE (2013). Plant sterol metabolism. Delta(7)-Sterol-C5-desaturase (STE1/DWARF7), Delta(5,7)-sterol-Delta(7)-reductase (DWARF5) and Delta(24)-sterol-Delta(24)-reductase (DIMINUTO/DWARF1) show multiple subcellular localizations in Arabidopsis thaliana (Heynh) L. PloS one.

[CR81] Sinclair AM, Trobacher CP, Mathur N, Greenwood JS, Mathur J (2009). Peroxule extension over ER-defined paths constitutes a rapid subcellular response to hydroxyl stress. Plant Journal.

[CR82] Sparkes I, Hawes C, Frigerio L (2011). FrontiERs: movers and shapers of the higher plant cortical endoplasmic reticulum. Curr Opin Plant Biol.

[CR83] Sparkes I, Runions J, Hawes C, Griffing L (2009). Movement and remodeling of the endoplasmic reticulum in nondividing cells of tobacco leaves. The Plant Cell.

[CR84] Sparkes IA, Ketelaar T, Ruijter NC, Hawes C (2009). Grab a Golgi: laser trapping of Golgi bodies reveals in vivo interactions with the endoplasmic reticulum. Traffic.

[CR85] Sparkes IA, Teanby NA, Hawes C (2008). Truncated myosin XI tail fusions inhibit peroxisome, Golgi and mitochondrial movement in tobacco leaf epidermal cells: a genetic tool for the next generation. Journal of Experimental Botany.

[CR86] Stefano G, Hawes C, Brandizzi F (2014). ER—the key to the highway. Curr Opin Plant Biol.

[CR87] Stefano G, Renna L, Brandizzi F (2014). The endoplasmic reticulum exerts control over organelle streaming during cell expansion. J Cell Sci.

[CR88] Stephenson JLM, Hawes CR (1986). Stereology and stereometry of the endoplasmic reticulum during differentiation in the maize root cap. Protoplasma.

[CR89] Takemoto D, Jones DA, Hardham AR (2003). GFP-tagging of cell components reveals the dynamics of subcellular re-organization in response to infection of Arabidopsis by oomycete pathogens. Plant J.

[CR90] Takemoto D, Jones DA, Hardham AR (2006). Re-organization of the cytoskeleton and endoplasmic reticulum in the Arabidopsis pen1-1 mutant inoculated with the non-adapted powdery mildew pathogen, Blumeria graminis f. sp. hordei. Mol Plant Pathol.

[CR91] Tamura K (2013). Myosin XI-i links the nuclear membrane to the cytoskeleton to control nuclear movement and shape in Arabidopsis. Curr Biol.

[CR92] Terasaki M (2013). Stacked endoplasmic reticulum sheets are connected by helicoidal membrane motifs. Cell.

[CR93] Uchiyama A, Shimada-Beltran H, Levy A, Zheng JY, Javia PA, Lazarowitz SG (2014). The Arabidopsis synaptotagmin SYTA regulates the cell-to-cell movement of diverse plant viruses. Frontiers in plant science.

[CR94] Ueda H (2010). Myosin-dependent endoplasmic reticulum motility and F-actin organization in plant cells. Proc Natl Acad Sci U S A.

[CR95] Underwood W, Somerville SC (2013) Perception of conserved pathogen elicitors at the plasma membrane leads to relocalization of the Arabidopsis PEN3 transporter Proc Natl Acad Sci U S A 110:12492–12497 doi:10.1073/pnas.1218701110 10.1073/pnas.1218701110 PMC3725069 23836668

[CR96] Van Bruaene N, Joss G, Van Oostveldt P (2004). Reorganization and in vivo dynamics of microtubules during Arabidopsis root hair development. Plant Physiol.

[CR97] van der Kant R, Neefjes J (2014). Small regulators, major consequences - Ca(2)(+) and cholesterol at the endosome-ER interface. J Cell Sci.

[CR98] Vidali L, Burkart GM, Augustine RC, Kerdavid E, Tuzel E, Bezanilla M (2010). Myosin XI is essential for tip growth in Physcomitrella patens. Plant Cell.

[CR99] Wang G et al. (2012) Opaque1 encodes a myosin XI motor protein that is required for endoplasmic reticulum motility and protein body formation in maize endosperm Plant Cell 24:3447–3462 doi:10.1105/tpc.112.101360 10.1105/tpc.112.101360 PMC3462643 22892319

[CR100] Wang P (2014). The plant cytoskeleton, NET3C, and VAP27 mediate the link between the plasma membrane and endoplasmic reticulum. Current Biology.

[CR101] Wozniak MJ, Bola B, Brownhill K, Yang YC, Levakova V, Allan VJ (2009). Role of kinesin-1 and cytoplasmic dynein in endoplasmic reticulum movement in VERO cells. J Cell Sci.

[CR102] Wright KM, Wood NT, Roberts AG, Chapman S, Boevink P, Mackenzie KM, Oparka KJ (2007). Targeting of TMV movement protein to plasmodesmata requires the actin/ER network: evidence from FRAP. Traffic.

[CR103] Yang L, Qin L, Liu G, Peremyslov VV, Dolja VV, Wei Y (2014). Myosins XI modulate host cellular responses and penetration resistance to fungal pathogens. Proc Natl Acad Sci U S A.

[CR104] Yokota E, Ueda H, Hashimoto K, Orii H, Shimada T, Hara-Nishimura I, Shimmen T (2011) Myosin XI-dependent formation of tubular structures from endoplasmic reticulum isolated from tobacco cultured cells, BY-2 Plant Physiology:doi: 10.1104/pp.1111.175018 doi:10.1104/pp.111.175018 10.1104/pp.111.175018 PMC3091044 21427277

[CR105] Yokota E (2009). An isoform of myosin XI is responsible for the translocation of endoplasmic reticulum in tobacco cultured BY-2 cells. Journal of Experimental Botany.

